# Genomic and metabolic differences between *Pseudomonas putida* populations inhabiting sugarcane rhizosphere or bulk soil

**DOI:** 10.1371/journal.pone.0223269

**Published:** 2019-10-03

**Authors:** Lucas Dantas Lopes, Alexandra J. Weisberg, Edward W. Davis, Camila de S. Varize, Michele de C. Pereira e Silva, Jeff H. Chang, Joyce E. Loper, Fernando D. Andreote

**Affiliations:** 1 Department of Soil Science, “Luiz de Queiroz” College of Agriculture, University of São Paulo, Piracicaba, SP, Brazil; 2 Department of Botany and Plant Pathology, Oregon State University, Corvallis, OR, United States of America; Universita Cattolica del Sacro Cuore, ITALY

## Abstract

*Pseudomonas putida* is one of 13 major groups of *Pseudomonas* spp. and contains numerous species occupying diverse niches and performing many functions such as plant growth promotion and bioremediation. Here we compared a set of 19 *P*. *putida* isolates obtained from sugarcane rhizosphere or bulk soil using a population genomics approach aiming to assess genomic and metabolic differences between populations from these habitats. Phylogenomics placed rhizosphere versus bulk soil strains in separate clades clustering with different type strains of the *P*. *putida* group. Multivariate analyses indicated that the rhizosphere and bulk soil isolates form distinct populations. Comparative genomics identified several genetic functions (GO-terms) significantly different between populations, including some exclusively present in the rhizosphere or bulk soil strains, such as D-galactonic acid catabolism and cellulose biosynthesis, respectively. The metabolic profiles of rhizosphere and bulk soil populations analyzed by Biolog Ecoplates also differ significantly, most notably by the higher oxidation of D-galactonic/D-galacturonic acid by the rhizosphere population. Accordingly, D-galactonate catabolism operon (*dgo*) was present in all rhizosphere isolates and absent in the bulk soil population. This study showed that sugarcane rhizosphere and bulk soil harbor different populations of *P*. *putida* and identified genes and functions potentially associated with their soil niches.

## Introduction

Rhizosphere is a soil compartment in close contact with plant roots and highly influenced by its rhizodeposits, such as exudates, lysates, mucilage, etc [1]. These compounds are scarce or absent in the bulk soil, *i*.*e*. the root-free soil [1]. Bulk soil is characterized by the accumulation of complex and recalcitrant C-sources present in decaying plant biomass and soil organic matter [2]. Microbial abundance and activity is typically higher in the rhizosphere than in bulk soil, and the two habitats have distinct microbiomes [3,4,5,6,7]. The rhizosphere microbiome can promote growth and support plants to resist biotic and abiotic stressors [8,9,10].

*Pseudomonas* spp. are bacteria commonly found in the rhizosphere microbiome and known for promoting plant growth and health [11,12,13]. The *Pseudomonas putida* group (called *P*. *putida* hereafter) contains at least 53 species that occupy diverse ecological niches [14]. Strains of *P*. *putida* perform many functions in the soil habitats, such as plant growth promotion (PGP) and bioremediation [15,16,17]. Among the PGP properties, strain BIRD-1 produces phytohormones and solubilizes phosphorus and iron [18]; strain FBKV2 increases drought stress tolerance in maize [19]; strain W619 produces phytohormones and catabolizes gamma-aminobutyric acid (GABA) [20]; and strain WCS358 induces systemic resistance in *Arabidopsis thaliana* [21]. On the other hand, strains DLL-E4, OUS-82 and DOT-T1E can degrade aromatic compounds present in contaminated soils [16,22,23,24]. For those reasons, understanding the ecology of soil *P*. *putida* is important to optimize its beneficial properties for a sustainable agriculture.

Considering the high genetic diversity of the *P*. *putida* group [14,17], comparing natural populations of *P*. *putida* inhabiting rhizosphere versus populations from other soil habitats (*i*.*e*. bulk soil) can identify distinctive features that resulted from their evolutionary process, providing new insights into niche adaptation. A bacterial population is defined as a set of co-existing individuals from the same or closely related species, highly clustered at the phylogenetic, genomic and phenotypic scales [25]. Distinct members of a bacterial population can evolve into ecotypes with specific niches [25,26,27,28]. We previously showed that the sugarcane rhizosphere and bulk soil select ecotypes differing in genome content within a *P*. *koreensis* population [29]. However, distinctions between sugarcane rhizosphere and bulk soil populations are unclear for other bacteria such as *P*. *putida*.

Therefore, here we used a population genomics approach to compare the genome sequences of 19 strains isolated from the rhizosphere or bulk soil of a sugarcane field in order to identify genes and functions enriched in each population. Physiological analyses were also performed to assess metabolic differences between rhizosphere and bulk soil isolates. The 19 strains were sequenced previously and classified in the *P*. *putida* group [30]. In the present study, we show a high degree of genomic and metabolic divergence between the strains isolated from the compared habitats, indicating that sugarcane rhizosphere and bulk soil harbor different populations of *P*. *putida*. We further identified some features potentially related to their distinct soil niches.

## Materials and methods

### Sampling and isolation

Soil samples were collected in 2014 from a sugarcane plantation of an agricultural experimental campus of the University of São Paulo, located in Piracicaba-SP, Brazil (22°69´S/47°64´W), managed for the past 10 years in a green-harvest cropping system. Twelve soil samples were obtained, six from the rhizosphere (1–2 mm soil adhering to roots) of different random plants in the field and six from bulk soil (root-free soil present between crop rows at a 0–10 cm depth) from different random points of the same sugarcane field (~300 g of soil in each point). Soil samples from rhizosphere or bulk soil were each homogenized into two composite samples prior to bacterial isolation. Approximately 50 g of soil from the composite samples was used to make serial dilutions in test tubes with 9 mL autoclaved water (1.0 g of soil in each tube). A total of 76 *Pseudomonas* spp. isolates were obtained by plating serial dilutions (10^−4^ to 10^−6^) of soil samples on the media Pseudomonas Agar Base (Oxoid, UK), selected based on fluorescence in UV light. The 76 isolates were submitted to whole genome sequencing, revealing a new biodiversity of *Pseudomonas* spp. in tropical soils [30]. Additional details of sampling, soil characterization and bacterial isolation are provided in Lopes et al. [30]. The 76 isolates were classified in the *P*. *fluorescens* (57) and *P*. *putida* (19) groups [30]. Here we analyzed all the 19 isolates from the *P*. *putida* group.

### Analyses of genome sequences and phylogenomics

The genomic DNA of the 76 isolates was sequenced in an Illumina HiSeq 3000 at the Center for Genome Research and Biocomputing (CGRB) of Oregon State University (OSU, USA) in a previous study, where information regarding genome sequencing, assembly and annotation are provided [30]. Accession numbers and main assembly results of the 19 isolates analyzed in the current study are available at S1 Table. A phylogenomic approach was performed in order to get the evolutionary relationship of the 19 isolates and known species classified in the *P*. *putida* group. For that, we firstly performed a multi-locus sequence analysis (MLSA) using a set of 100 gene sequences previously used to depict the evolutionary history of the *Pseudomonas* genus [14]. Ninety-nine reference protein sequences from *Pseudomonas aeruginosa* strain PAO1 were used as queries to retrieve homologous sequences from NCBI GenBank database on June 6th, 2018, using AutoMLSA v. 1.0 with the default parameters [31]. These genes represent the 100 single-copy core genes from Hesse et al. [14] minus *ampG* (PA4393), which was not present in all 19 strains.

A total of 533 genome sequences containing the 99 gene sequences and tagged as *Pseudomonas* spp., including type and reference strains of many *Pseudomonas* groups, was retrieved from NCBI GenBank database using autoMLSA v. 1.0 software [31]. This approach allowed for the inclusion of 15 type strains of species within the *P*. *putida* group [14]. Two type strains of the *P*. *putida* group (*P*. *donghuensis* and *P*. *coleopterum*) were not included because genome sequences were unavailable [14]. Mafft v. 7.271 (L-ins-I algorithm) was used to align the sequences of each genome [32]. AutoMLSA v. 1.0 was used for concatenation of the aligned sequences and RAxML v. 8.2.11 was used to construct a maximum likelihood phylogenetic tree (100 maximum likelihood search trees, autoMRE bootstrap replicates) [31,33]. GTRGAMMA substitution model was used for the tree [34]. TreeCollapseCL4 was used to collapse branches with less than 50% bootstrap support into polytomies [35]. The tree was annotated and visualized using the ItoL platform [36].

In parallel, we performed another phylogenomic analysis based on genome BLAST distance phylogeny (GBDP), an approach that compares the whole genome sequences of strains [37,38]. For that, we used the Genome to Genome Distance Calculator (GGDC) online platform (https://ggdc.dsmz.de/) with the recommended alignment tool (BLAST+) to generate the BLAST distances between the genome sequences of the19 isolates and 15 type strains of the *P*. *putida* group, whose assembly accession numbers are provided in S2 Table [38]. In addition, we estimated the digital DNA-DNA hybridization (dDDH) between these 34 genome sequences in the same platform. The digital DDH is estimated based on the distance values calculated by GGDC using a generalized linear model (GLM) inferred from an empirical reference dataset comprising real DDH values and genome sequences [38].

The resulting BLAST distance values calculated by the recommended formula (identities/HSP length), as well as the dDDH values between each of the 34 genome sequences were organized into distance matrices. The genome BLAST distance matrix was exported to Primer-6 software, where a cluster analysis based on UPGMA was performed to generate a dendrogram/tree of GBDP [39,40]. The dDDH matrix was exported to the Heatmapper online platform (http://www.heatmapper.ca/), where a heatmap was generated to illustrate the genomic relationship between the 19 isolates and type strains using this third approach, as well as to infer on the taxonomic affiliation and number of possible species within each population.

### Population structure and comparative genomics analyses

Putative orthologues present in the 19 *P*. *putida* genome sequences were inferred by clustering genes using the Get Homologues software with the OrthoMCL algorithm [41,42]. Sequences were grouped in gene clusters using the default settings of Get Homologues, which is all-versus-all BLASTp with a minimum coverage of 75% and an e-value cutoff of 1x10^-5^. A matrix containing the abundance of each gene cluster found in the isolates was generated. Gene clusters present in isolates of both habitats or specific to the isolates of each habitat were detected using the same software. The same approach and settings were used to analyze the genomic variability between the isolates of each habitat separately, in order to identify the number of gene clusters specific to each strain and the core/accessory genome within each population.

Multivariate analyses were performed to assess the differences in genome content between the isolates from rhizosphere or bulk soil. Firstly, the matrix of gene clusters was exported to Primer-6 software, where data was normalized (log transformation) and analyzed by non-metric multidimensional scaling (NMDS) using the Bray-curtis index to check the ordination of bulk soil and rhizosphere samples, while analysis of similarity (ANOSIM) was applied to test the statistical significance of those differences [40]. Then, population structure was analyzed by discriminant analysis of principal components (DAPC) using the adegenet package in R software based on the same matrix of gene clusters [43,44].

Aiming to compare the genetic functions found between the rhizosphere and bulk soil populations, we used InterProScan 5 software to identify conserved protein domains in the translated amino acid sequences of each gene cluster based on Pfam and TIGRFAM protein databases [45]. Then, GO-terms were attributed to each inferred function based on the identification of conserved domains in the amino acid sequences. The three categories of GO-terms (molecular function, cellular component and biological process) were comprised in the analysis. The abundance of GO-terms in each of the 19 genome sequences was organized into a matrix. GO-terms specific to the rhizosphere or bulk soil strains (present in all rhizosphere isolates and absent in all bulk soil isolates, and *vice-versa*) were identified. Additionally, the GO-terms matrix was exported to STAMP software, where a statistical approach using Welch’s t-test with Bonferroni *P*-value correction was performed to detect the functions (GO-terms) significantly different between the rhizosphere and bulk soil strains [46].

### Analyses of the metabolic profile

The metabolic profile of the 19 isolates was assessed using BIOLOG Ecoplates, which contains 31 wells with different single C-sources [47]. Purified cultures of each isolate were grown in Luria-Bertani (LB) media for 24 hours at 28°C [48]. Cells were washed twice with autoclaved MQ water, followed by adjusting the optical density of all cultures before inoculating 150 μL in the BIOLOG plates, which were immediately incubated at 28°C. Absorbance readings were performed after 24 hours in 600 nm. Each isolate culture was inoculated in two different plates, aiming to both use replicates and reduce potential bias associated with individual plates.

The absorbance tables of the BIOLOG analyses were exported to Primer-6 software, where data was normalized (log transformation) and NMDS was performed using the Bray-Curtis index to get the ordination of samples based on the metabolic profile of the isolates [40]. SIMPER analysis was performed to identify the C-sources most contributing to the differences between bulk soil and rhizosphere samples in the same software. Data was also exported to CANOCO 4.5, where a principal components analysis (PCA) biplot was performed to analyze the correlation of samples (isolates) and variables (C-sources) [49]. The C-sources were individually compared between rhizosphere and bulk soil isolates by Tukey’s pairwise comparison test on Past software [50]. In addition, a Pearson correlation test between the 31 C-sources was performed using the same software.

## Results and discussion

### Phylogenomic distinction between rhizosphere and bulk soil strains

**Fig 1:**
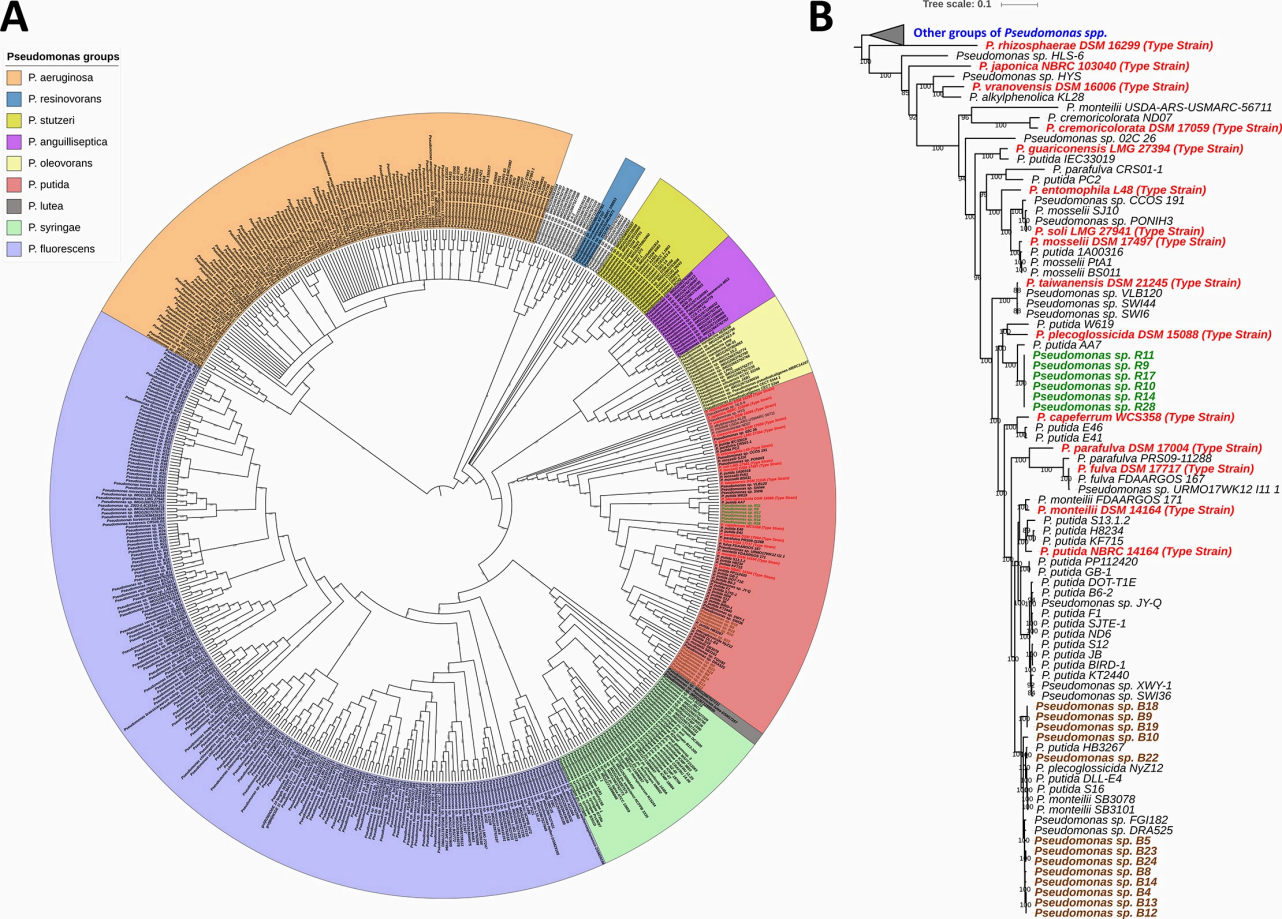
Maximum likelihood phylogenetic tree of the isolates. A) Ninety-nine orthologous gene sequences present in single copies in all 19 isolates of this study, 15 type strains of the *P*. *putida* group and 518 strains of other eight *Pseudomonas* groups were aligned, concatenated and used to generate a MLSA phylogenetic tree, which is rooted in the *P*. *aeruginosa* group. B) Zoom in of the *P*. *putida* group. The tree was rooted in the *P*. *putida* group and the clades containing strains from the other groups of *Pseudomonas* spp. were collapsed and used as outgroup. The isolates assessed in this study are labeled in green font (rhizosphere) or brown font (bulk soil); the reference strains are labeled in black font; and the type strains of the *P*. *putida* group are labeled in red font. The bootstrap values are shown below each branch (100 bootstrap tests).

The genome sequences of the 19 *P*. *putida* isolates (6 from bulk soil and 13 from rhizosphere) were previously described showing an average genome size of 5.85 Mbp, which is consistent to complete genome sequences of closely related strains of the *P*. *putida* group [30]. To determine if the rhizosphere and bulk soil isolates are different ecotypes of the same population or comprise two distinct populations, we first used a phylogenomics approach. Ninety-nine putative orthologues present in single copies in the 19 *P*. *putida* genome sequences, in 15 type strains of species classified in the *P*. *putida* group, and in other 518 genome sequences from strains of the *Pseudomonas* genus available in the Genbank database, were aligned, concatenated and a maximum-likelihood phylogenetic tree was generated (Fig 1A). The 552 strains used in this MLSA were comprised in nine of the 13 groups where *Pseudomonas* spp. are currently classified [14]. The MLSA tree using this higher set of gene markers (99) separated the rhizosphere and bulk soil isolates into distinct phylogenetic clades within the *P*. *putida* group (Fig 1B), confirming previous MLSA that used a few gene markers [30].

However, the present MLSA tree also allowed a better taxonomic comparison of the 19 isolates with known species in the *P*. *putida* group by including type strains. The rhizosphere strains were closely related between each other and more related to the type strain of *P*. *plecoglossicida* than to the type strains of the other 14 species analyzed. In contrast, the bulk soil strains showed a higher phylogenetic diversity and were more closely related to the type strains of *P*. *monteilii* and *P*. *putida* (Fig 1B). Three type strains are phylogenetically placed between the bulk soil and rhizosphere strains using this approach (*P*. *capeferrum*, *P*. *parafulva* and *P*. *fulva*). Two type strains classified in the *P*. *putida* group were not included in this analysis (*P*. *donghuensis* and *P*. *coleopterum*), but previous studies showed that they cluster to *P*. *rhizosphaerae* and *P*. *vranovensis* [14], which are very distant to the species clustering to our 19 isolates (Fig 1B). Besides the comparison with type strains, it is noteworthy that the rhizosphere strains clustered to the PGP strain W619 [20], whereas the bulk soil strains clustered to the aromatic catabolizing strain DLL-E4 [23], which could be associated with different lifestyles of strains from each habitat (Fig 1B). It is possible that the rhizosphere isolates participate in plant-microbe interactions, while the bulk soil isolates could be more efficient in degrading aromatic compounds from crop residues commonly found in bulk soil, such as lignin [51].

**Fig 2:**
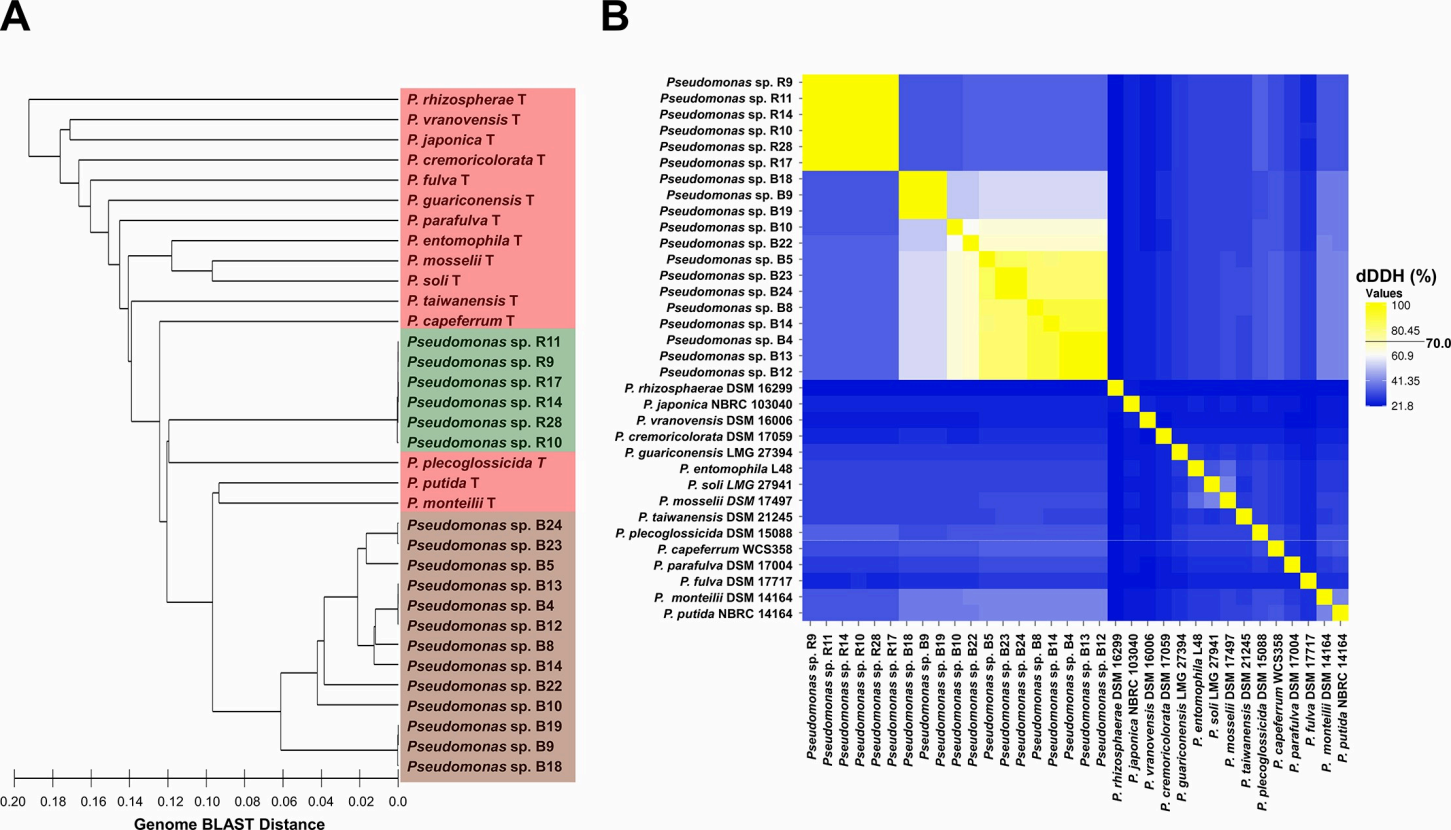
Whole-genome comparison between isolates and type strains of the *P*. *putida* group. A) Genome BLAST distance phylogeny (GBDP) clustering the 19 isolates and 15 type strains by UPGMA. *P*. *rhizosphaerae* DSM 16299 was used as outgroup. Labels of the type strains, rhizosphere isolates and bulk soil isolates are in the hatched areas colored in pink, green and brown, respectively. Type strains are labeled with a “T” after the name of the species B) Heatmap of the digital DNA-DNA hybridization (dDDH) between these 34 strains. Strains sharing more than 70% dDDH are colored in yellow, while strains sharing less than 70% are colored in beige, white or blue.

Our second phylogenomic approach analyzed the whole genome sequences of the isolates and the 15 type strains previously mentioned based on BLAST distances. Whole genome-based approaches have been shown to be powerful in reconstructing the phylogeny of the *P*. *fluorescens* species complex [52]. The GBDP tree agreed with MLSA by clustering the rhizosphere strains to the species *P*. *plecoglossicida*, despite a high distance was observed between them (Fig 2A). Similarly, the bulk soil strains also clustered to the *P*. *monteilii* and *P*. *putida* species, corroborating the MLSA tree. However, the GBDP tree differed from MLSA in not placing the type strains of *P*. *fulva*, *P*. *parafulva* and *P*. *capeferrum* between the rhizosphere and bulk soil isolates, besides changing the clustering of some type strains (Fig 2A).

To get the taxonomic status of the 19 strains, we analyzed the dDDH between each other and compared with the type strains. Interestingly, none of the 19 isolates showed more than 70% dDDH to any of the 15 type strains, including the closest species *P*. *plecoglossicida*, *P*. *putida* and *P*. *monteilii*, which suggests that the 19 isolates are from unknown new species (Fig 2B). The rhizosphere isolates ranged from 99.8 to 100% dDDH between each other, indicating that they are the same species. On the other hand, the bulk soil isolates ranged from 52.2 to 100% dDDH, indicating that more than one species (possibly four) are comprised in this population (Fig 2B). However, all the bulk soil strains showed higher dDDH values between each other than when comparing to rhizosphere strains, whose average dDDH was 34.9%.

### Rhizosphere and bulk soil harbor different populations of *P*. *putida* according to genome content

**Fig 3:**
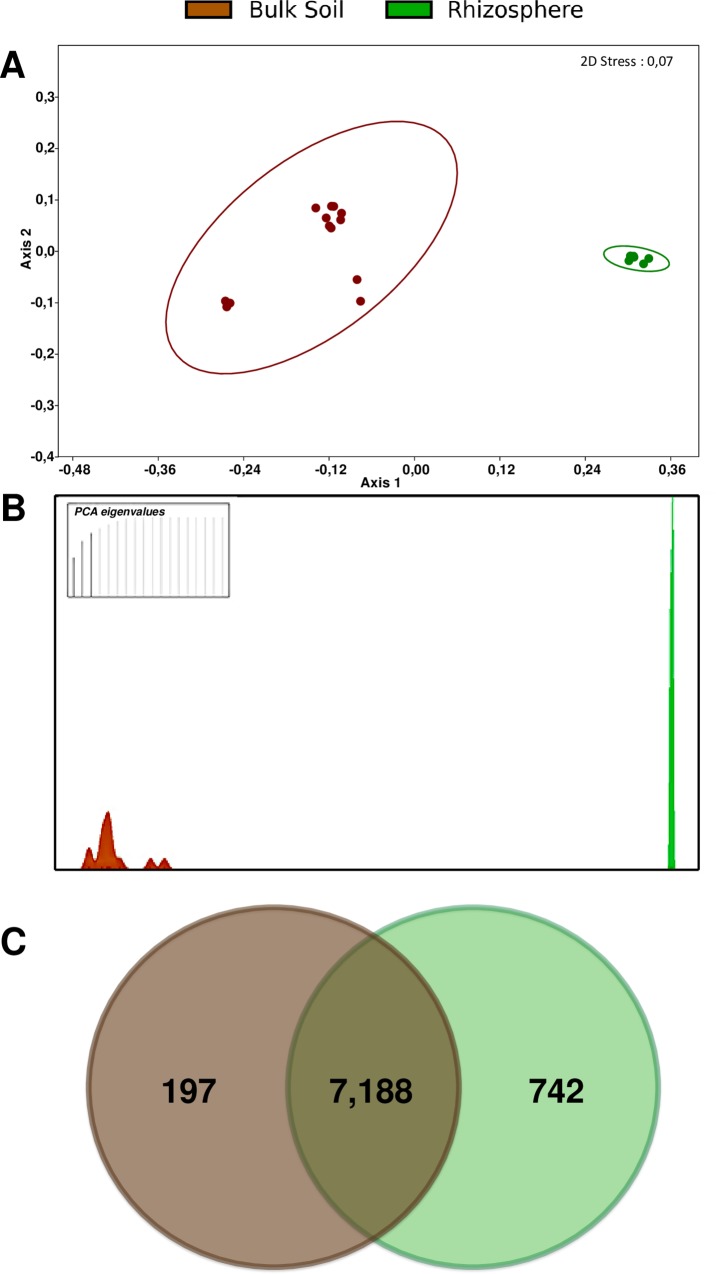
Analyses of all gene clusters present in the 19 genome sequences. A-B) Multivariate statistical analyses comparing the populations of each habitat. A) NMDS showing the different clustering of rhizosphere and bulk soil isolates; B) DAPC indicating that rhizosphere and bulk soil isolates form distinct populations. Only 3 PCA eigenvalues were needed to discriminate the populations (top left). C) Numbers of gene clusters shared or specific to rhizosphere and bulk soil populations.

After the phylogenomic analyses, we then investigated the gene content of the rhizosphere and bulk soil isolates. Putative orthologues were inferred by Get Homologues software and grouped sequences in gene clusters. A total of 8,127 individual gene clusters were detected among the 19 isolates. The 8,127 detected gene clusters were then analyzed by multivariate analyses in order to assess the degree of genomic divergence between the rhizosphere and bulk soil isolates. A non-metric multidimensional scaling (NMDS) confirmed that the isolates from the different habitats form distinct groups according to the genome content (Fig 3A), supported by analysis of similarity (ANOSIM, *P*<0.01). Discriminant analysis of principal components (DAPC), a multivariate approach designed to analyze population structure [43], showed a high degree of separation between rhizosphere and bulk soil isolates, even when considering only 3 PCA eigenvalues (Fig 3B). In addition, Get Homologues analysis identified almost a thousand (939) gene clusters that are exclusive to the rhizosphere (742) or bulk soil (197) isolates (Fig 3C).

Regarding the genomic variability between the strains within each population, it was observed a higher diversity in the bulk soil than in the rhizosphere strains. Rhizosphere isolates ranged from 1 to 18, while bulk soil isolates ranged from 0 to 336 exclusive (strain-specific) gene clusters (Table 1). The high phylogenetic and genomic proximity of the rhizosphere isolates suggests that some of them are possibly the same strain, although specific gene clusters were observed in each one. For those reasons and because some bulk soil strains are from different species (Fig 2B), the rhizosphere population showed a lower accessory genome (cloud + shell) and higher core genome than the bulk soil population (Table 1).

**Table 1:** Number of gene clusters exclusive to each strain (strain-specific), shared with a maximum of one other strain (cloud), shared with more than two strains and less than 95% of strains (shell), shared with more than 95% of strains (soft core), shared with 100% of strains (core), and total of gene clusters in each population.

	Rhizosphere population						Bulk Soil population												
	R9	R10	R11	R14	R17	R28	B4	B5	B8	B9	B10	B12	B13	B14	B18	B19	B22	B23	B24
Strain-specific	4	18	3	1	5	4	6	187	160	7	336	1	0	166	6	3	288	16	11
Cloud (< = 2)	8	20	7	7	7	6	10	222	187	17	361	49	49	220	14	11	358	280	277
Shell (>2) (<95%)	11	6	14	14	16	4	1023	845	803	844	612	1027	1025	839	844	845	572	789	786
Soft core (>95%)	5209	5204	5210	5210	5210	5209	4052	4033	4045	4052	4015	4052	4053	4049	4050	4053	3963	4051	4050
Core (100%)	5197						3882												
Total	5273						7629												

Together, the results showed a high genomic divergence between the rhizosphere and bulk soil isolates, indicating that the *P*. *putida* strains here analyzed comprise two independent populations. Therefore, this is a different case compared to previous results reported by our group for a *P*. *koreensis* population, where the isolates from rhizosphere and bulk soil were not in distinct populations, but in different ecotypes within a common population [29]. The rhizosphere and bulk soil ecotypes of *P*. *koreensis* do not have a clear phylogenetic distinction and showed only slight differences in genome content [29]. Results also indicated that a *P*. *putida* population of closely related individuals inhabits sugarcane rhizosphere, while a more diverse population of *P*. *putida* live in bulk soil. The lower genetic diversity of *P*. *putida* in the rhizosphere is consistent with studies assessing microbial communities, where bacterial species diversity is usually lower in the rhizosphere compared to bulk soil, despite the former generally has a higher microbial abundance and activity [3,6,9,53,54].

### Genetic functional differences between rhizosphere and bulk soil populations

The phylogenetic and genomic distinction observed in the previous sections between the *P*. *putida* populations isolated from rhizosphere versus bulk soil indicates the possibility that many traits evolved in each population for niche adaptation. Thus, we set out to identify these genetic functions in order to improve our understanding regarding the ecology of each population. Conserved protein domains were identified and GO-terms were attributed to each gene cluster detected by Get Homologues software, generating a matrix of functions (GO-terms) among the 19 isolates. A total of 1,378 GO-terms were attributed to the set of gene clusters. The number of hits for each GO-term ranged from 1 to >500 in a single strain, reflecting more specific and more generalist functions, respectively.

**Fig 4:**
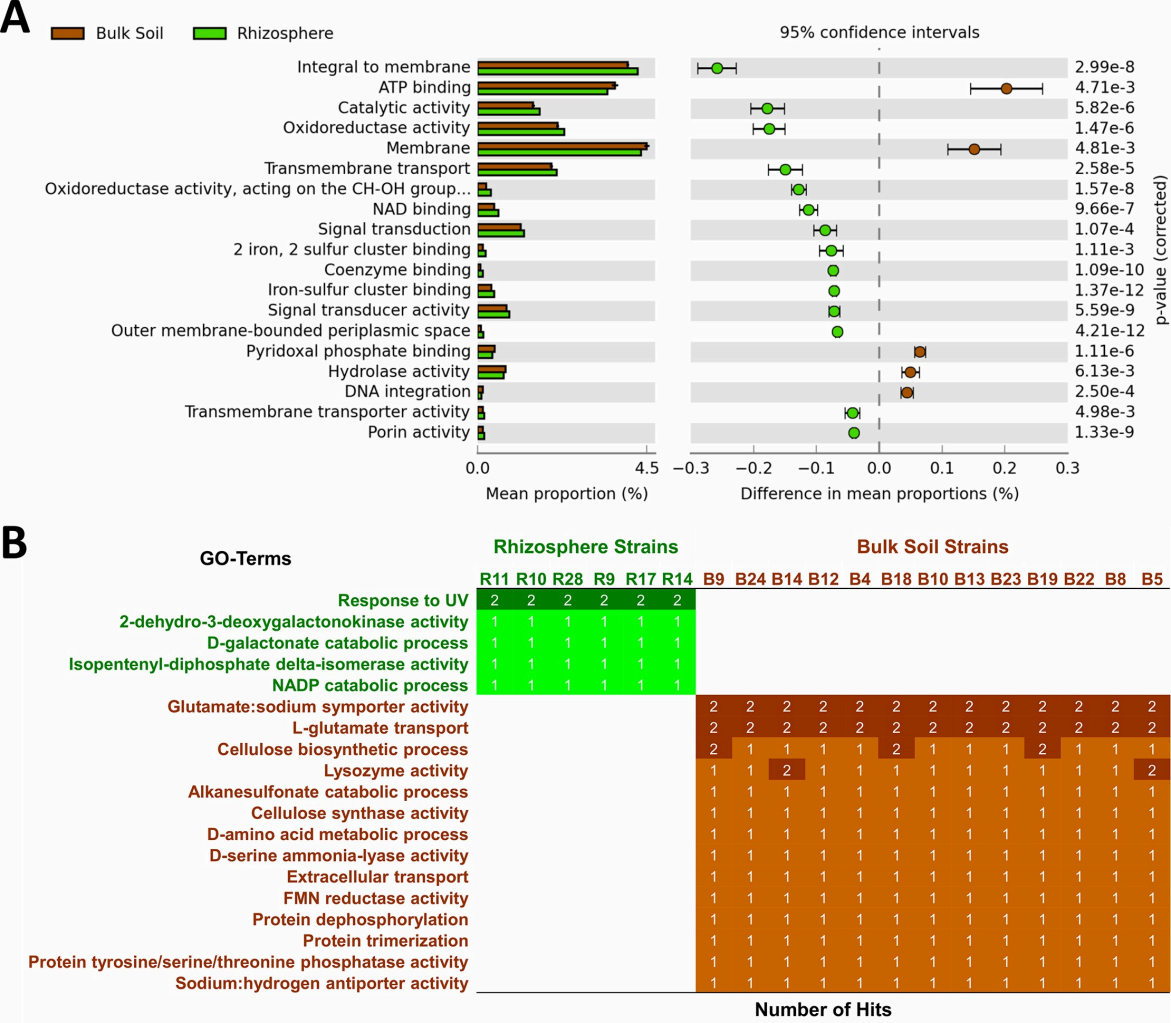
Differences in GO-terms between rhizosphere and bulk soil populations. A) Statistical analysis based on Welch’s t-test and Bonferroni *P*-value correction showing the major GO-terms enriched in the rhizosphere or bulk soil populations (*P*<0.05). GO-terms with the higher differences in hits proportion (>0.06%) between rhizosphere and bulk soil populations are shown in the graph. B) All GO-terms (19) showing total presence in a population and total absence in the other population (rhizosphere versus bulk soil). Number of hits of each GO-term in each strain is shown. Rhizosphere strains and GO-terms exclusive to all rhizosphere isolates are labeled in green, while bulk soil strains and GO-terms exclusive to all bulk soil isolates are labeled in brown.

To identify functions enriched in the populations of rhizosphere or bulk soil, we used a statistical approach similar to that applied for the *P*. *koreensis* population in our previous study [29]. We used Welch’s t-test with the Bonferroni *P*-value correction (the most restrictive method) to compare the populations. Results revealed a total of 161 functions (GO-terms) with significantly different abundances between the rhizosphere and bulk soil populations (*P*<0.05) (S1 Fig). Sixteen major functions differed more than 0.06% in the proportion of hits between the rhizosphere and bulk soil populations (Fig 4A). These more generalist GO-terms are a product of several genes and their different proportions in rhizosphere versus bulk soil strains reflect the distinct abundance of some genes contributing to these functions in the analyzed genomes (Fig 4A).

Among these major functions, those associated with transmembrane transport and signal transduction (Integral to membrane, Transmembrane transport, Signal transduction and Signal transducer activity) are enriched in the rhizosphere population, suggesting that genes functioning in communication with the plant host and/or the rhizosphere microbiome are more frequent in this population, despite present in both (Fig 4A). One of the GO-terms directly involved in bacterial signaling and enriched in the rhizosphere population is Protein histidine kinase activity (S1 Fig). Histidine kinases are transmembrane sensor kinase proteins participating in the two-component system, one of the most important methods of signal transduction in prokaryotes [55]. The intricate molecular microbe-microbe and plant-microbe interactions in the rhizosphere are mediated by the release and absorption of chemical compounds resulting in cell signaling and communication [56], which possibly explains the enrichment of those genes in the rhizosphere population.

A similar number of catabolic functions are enriched in each population, but more hydrolase activity GO-terms are enriched in the bulk soil population, while more oxidoreductase activity GO-terms are enriched in the rhizosphere population (S1 Fig). On the other hand, more anabolic functions, including biosynthesis of cellulose, fatty acids and phospholipids, are enriched in the bulk soil than in the rhizosphere population. One hypothesis for the significantly higher abundance of these GO-terms in the bulk soil strains could be a higher demand of more resistant cell membranes, leading to drought tolerance [57], since water stress is higher in bulk soil than in rhizosphere [58].

Genes with predicted functions in phosphorus acquisition and transport are mostly enriched in the rhizosphere population (S1 Fig). Tropical soils are often characterized by a decreased availability of phosphate to plants, which is essential for their nutrition and growth [59]. Genes related to transport of inorganic phosphate and organic acids that solubilize phosphate attached to mineral surfaces are significantly higher in the rhizosphere population. Our previous study assessing a *P*. *koreensis* population also identified more genes related to phosphate acquisition in the rhizosphere compared to the bulk soil ecotypes [29]. Other functions enriched in the rhizosphere population are associated with cell outer membrane, cell adhesion, binding, and lipopolysaccharide (LPS) biosynthesis (S1 Fig). LPS are compounds integrating biofilms, which are important for rhizosphere colonization [60,61,62]. Furthermore, the rhizosphere population also has more functions associated with ion binding and transport, suggesting a higher capacity to capture inorganic compounds (S1 Fig).

Then, we analyzed the matrix of GO-terms to identify the genetic functions present only in the rhizosphere or in the bulk soil populations, *i*.*e*. the most drastic differences. Five GO-terms were specific to the rhizosphere population and 14 GO-terms were present only in the bulk soil population (Fig 4B). This was not expected because more gene clusters are specific to the rhizosphere population (Fig 3C). A possible explanation is that several of the gene clusters specific to the rhizosphere population are functionally redundant to gene clusters present in the bulk soil population, *i*.*e*. many genes present only in the rhizosphere isolates code for functions categorized in the same GO-terms that are also present in bulk soil isolates. On the other hand, few hits were observed for those 19 GO-terms exclusive to each population, indicating that they are a product of specific genes (Fig 4B).

Among the specific functions, the rhizosphere population exclusively has two GO-terms associated with D-galactonic acid catabolism (2-dehydro-3-deoxygalactonokinase and D-galactonate catabolic process), while the bulk soil population exclusively has two GO-terms associated with biosynthesis of bacterial cellulose (Cellulose biosynthetic process and Cellulose synthase activity) (Fig 4A). Cellulose is a component of the extracellular matrix of bacterial cells [63,64], which helps bacteria to survive in the harsh conditions of bulk soil, such as the high water stress, as previously discussed [57,58]. Despite extracellular matrix was also shown to be important for biofilm formation and PGP function of rhizosphere bacteria [60], our results suggest that cellulose might be an essential component of the extracellular matrix of the bulk soil strains here analyzed. D-galactonate is a uronic acid resulting from the oxidation of D-galactose, a sugar present in root secretion and possibly used for bacterial nutrition in the rhizosphere habitat [65,66].

Other GO-terms exclusive to the rhizosphere and bulk soil populations were response to UV light and glutamate transport, respectively (Fig 4B). The populations containing these GO-terms showed two hits for each strain. The reason for the presence of genes associated with response to UV light in the rhizosphere population is unclear. It is possible that phototaxis could attract these bacteria to colonize rhizosphere, since roots are located in superficial soil layers. However, to date no study indicated this type of attraction for bacterial colonization in the rhizosphere. On the other hand, the presence of genes associated with glutamate transport (and glutamate:sodium symport) in the bulk soil population might be associated with a higher ability of these strains to degrade the complex compounds of soil organic matter and plant residues, since sodium glutamate was shown to act in the cometabolic transformation of phenols in *P*. *putida* [67].

### Metabolic differences between populations is mainly determined by use of D-galactonic and D-galacturonic acids

**Fig 5:**
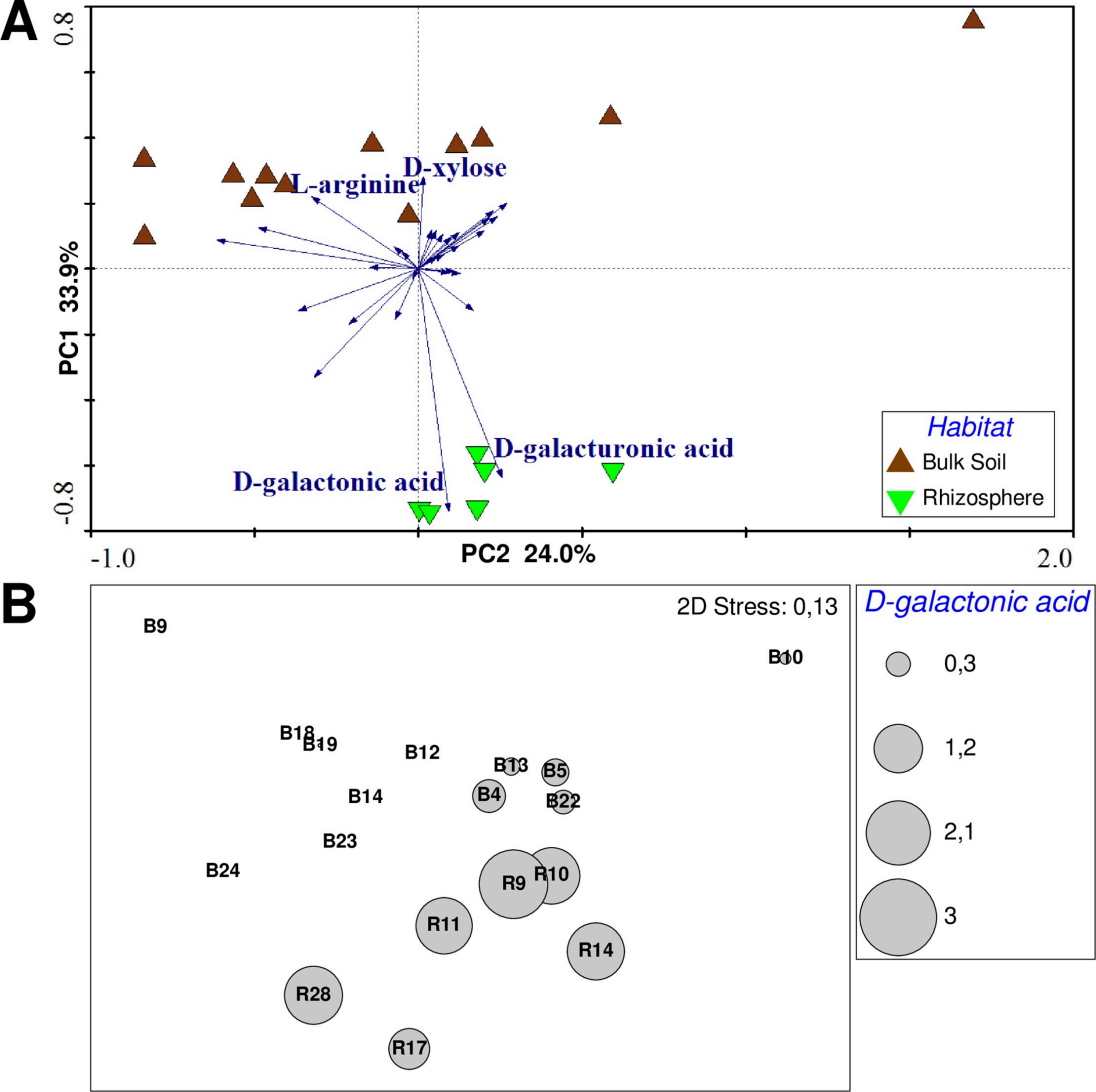
Metabolic profile of the rhizosphere and bulk soil isolates. A) PCA biplot showing the different clustering of the isolates from each habitat, as well as the correlation of four C-sources and the bulk soil or rhizosphere samples. These four C-sources were significantly different between rhizosphere and bulk soil (Tukey test, *P*<0.05); B) NMDS showing the different levels of oxidation of D-galactonic acid in the rhizosphere and bulk soil isolates. The size of the circles are proportional to the absorbance values for D-galactonate oxidation in each strain (B = bulk soil strains, R = rhizosphere strains).

To verify whether the genomic differences are also observed at the physiological level, we next assessed the metabolic profile of the 19 isolates. The metabolic profile of the 19 isolates was analyzed using BIOLOG ecoplates [47]. Multivariate analyses (biplot PCA and NMDS) compared the pattern of oxidation of the 31 individual C-sources by each isolate and displayed the different ordination of bulk soil and rhizosphere samples (Fig 5A and 5B). ANOSIM confirmed that the physiological profiles of rhizosphere and bulk soil populations were significantly different (*P* = 0.022). D-galactonic acid and D-galacturonic acid were the C-sources that contribute most to the metabolic differences between the rhizosphere and bulk soil populations as revealed by SIMPER analysis, with a 8.88% and 8.45% contribution, respectively. All rhizosphere isolates were able to use these compounds, while most bulk soil isolates lack this trait. The few bulk soil isolates capable of using D-galactonate had an average oxidation 10 times lower than the rhizosphere isolates (Fig 5B). Only four C-sources showed individual significant differences (*P*<0.05) between the compared populations: D-galatonic/D-galacturonic acid, which were more oxidized by the rhizosphere isolates, and L-arginine/D-xylose, which were more oxidized by the bulk soil isolates (Fig 5A).

D-galacturonic acid is the main component of pectin in plant cell walls and was detected in the root exudates of some plants [68,69]. On the other hand, D-galactonic acid is a product from the catabolism of D-galactose, a sugar also present in pectin and found in root exudates/mucilage [65,66,68]. The higher oxidation of both molecules indicates the higher efficiency of the rhizosphere population to consume the pectic compounds secreted in the rhizosphere.

Catabolism of galactosides and galacturonates were previously suggested to play a role in the symbiotic relationship among rhizobia and plants [65,70]. However, the relevance of these organic compounds for the colonization of rhizosphere by mutualistic bacteria such as *Pseudomonas* spp. remains unclear. Our study shows genomic and phenotypic evidence that utilization of D-galactonic and D-galacturonic acid is a characteristic of the assessed rhizosphere population, which probably poses an ecological advantage to colonize this habitat compared to the bulk soil population. In a previous study analyzing the microbial community of the same soil samples, we also found higher oxidation levels of D-galactonic/D-galacturonic acid, as well as a higher abundance of the polygalacturonase gene (from metagenome prediction analysis) in the rhizosphere compared to the bulk soil microbiome [7], suggesting that utilization of pectic compounds can be a selecting factor for rhizosphere colonization at the community level as well.

Finally, we mined the genomes to identify the genes associated with D-galactonate and D-galacturonate utilization. Regarding the catabolism of D-galactonate, we found the genes comprising the galactonate operon *dgoKADT*, which is known to function in D-galactonate utilization by *Escherichia coli* [71]. We identified *dgoT* (galactonate transporter), *dgoD* (dehydratase), *dgoK* (kinase) and *dgoA* (aldolase) genes in the six rhizosphere isolates. In contrast, these four genes are absent in the 13 bulk soil isolates, which resulted in the presence or absence of the GO-terms associated with D-galactonate utilization in the rhizosphere and bulk soil populations, respectively (Fig 4B). Thus, the presence of this operon potentially caused the higher oxidation levels of D-galactonate by the rhizosphere population. However, it is necessary to experimentally verify the activity of these genes.

With respect to D-galacturonate catabolism—the monosaccharide directly released from pectin backbone degradation—we did not find genes associated with any of the three described pathways in the 19 genome sequences [72]. Nevertheless, the physiological analysis showed that the rhizosphere isolates are able to use D-galacturonic acid in high amounts, implying that they must have another unknown pathway for D-galacturonate catabolism (Fig 5B). One hypothesis is that D-galacturonate is somehow converted to D-galactonate, which is then catabolized in the pathway encoded by *dgo* genes. This suggestion is based on the significant correlation between D-galactonic and D-galacturonic acid oxidation in BIOLOG analysis (*r* = 0.92; *P* = 5.95x10^-8^), the highest correlation observed between the 31 C-sources analyzed. Alternatively, a complete new pathway for D-galacturonate utilization might exist. Future analyses are needed to elucidate the metabolism of D-galacturonate in *P*. *putida*.

Regarding the bulk soil population, it is noteworthy that our previous study with a *P*. *koreensis* population also showed a higher efficiency in xylose consumption in the bulk soil isolates, a feature associated with the presence of *xut* genes [29]. The bulk soil isolates of *P*. *putida* here assessed lack *xut* genes, but are still able to use xylose in higher amounts than their rhizosphere counterparts (Fig 5A), indicating that other unknown pathways might exist for this function and that xylose utilization could also be a selecting factor for strains of the *P*. *putida* group inhabiting bulk soil.

## Conclusions

The present results show that rhizosphere and bulk soil of a sugarcane field in Brazil harbor distinct populations of *Pseudomonas putida* differing at the phylogenetic, genomic, metabolic and gene levels. Some genetic functions are exclusive to each population, including biosynthesis of bacterial cellulose in the bulk soil population and catabolism of D-galactonic acid in the rhizosphere population, which may be important for their respective niches in soil. Physiological analyses confirmed the higher oxidation levels of D-galactonic and D-galacturonic acids by the rhizosphere population. Several other functions are differently distributed between the rhizosphere and bulk soil populations, despite present in both. The genomic and metabolic divergence of these populations might have resulted from the differential selective pressures posed by the bulk soil versus rhizosphere habitats.

## Supporting information

S1 Fig All 161 GO-terms significantly different between the populations. Statistical analysis based on Welch’s t-test with Bonferroni *P*-value correction for detecting the GO-terms significantly enriched in the rhizosphere and bulk soil populations (*P*<0.05). Numbers refer to predicted functions of the GO-terms discussed along the manuscript: 1) Catabolism; 2) Biosynthesis; 3) Membrane transport/signaling; 4) Ion binding/transport; 5) Phosphorus cycle/acquisition; 6) Outer membrane/Cell adhesion; 7) DNA/Horizontal gene transfer (HGT); 8) Others.
(PDF)

S1 Table Accession numbers, n° of contigs and predicted genome sizes of the 19 *P*. *putida* genome sequences available in the Genbank/DDBJ/ENA databases. (DOCX)

S2 Table Accession numbers and predicted genome sizes of the 15 type strains within the *P*. *putida* group available in NCBI Genbank database. (DOCX)
