# Supplementary figures and images for: Genomic and metabolic differences between Pseudomonas putida populations inhabiting sugarcane rhizosphere or bulk soil
Source: PLoS One. 2019 Oct 3;14(10):e0223269. doi: 10.1371/journal.pone.0223269 (PMC6776310; doi:10.1371/journal.pone.0223269)

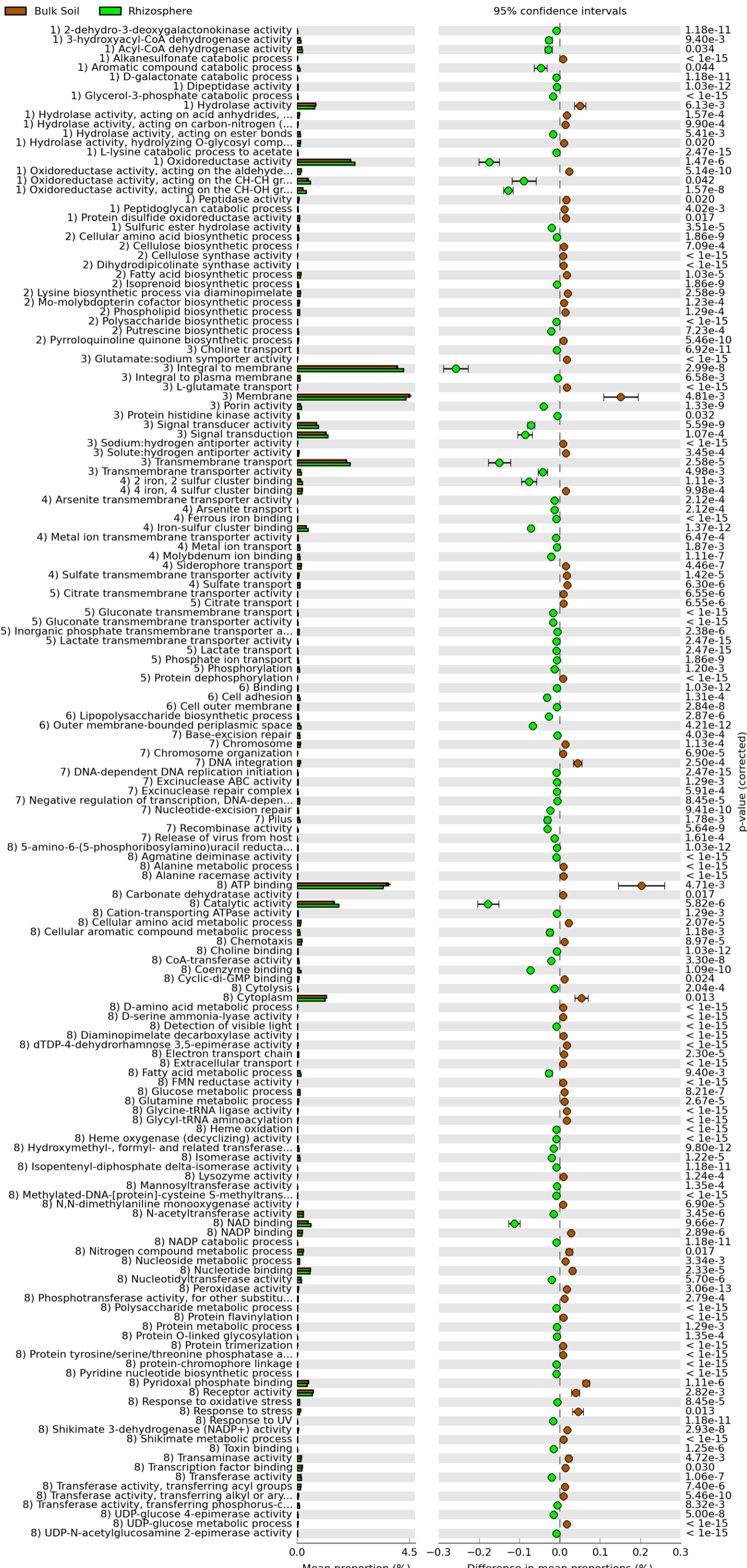

Supplement: S1 Fig — Statistical analysis based on Welch’s t-test with Bonferroni P-value correction for detecting the GO-terms significantly enriched in the rhizosphere and bulk soil populations (P<0.05). Numbers refer to predicted functions of the GO-terms discussed along the manuscript: 1) Catabolism; 2) Biosynthesis; 3) Membrane transport/signaling; 4) Ion binding/transport; 5) Phosphorus cycle/acquisition; 6) Outer membrane/Cell adhesion; 7) DNA/Horizontal gene transfer (HGT); 8) Others. (PDF) [file pone.0223269.s001.pdf]
