# Supplementary material for: Genomic and metabolic differences between Pseudomonas putida populations inhabiting sugarcane rhizosphere or bulk soil
Source: PLoS One. 2019 Oct 3;14(10):e0223269. doi: 10.1371/journal.pone.0223269 (PMC6776310; doi:10.1371/journal.pone.0223269)
Supplement: S1 Table — (DOCX) [file pone.0223269.s002.docx]

| SUBID | BioSample | Accession | Organism | Nº of contigs | Genome Size (Mbp) |
| --- | --- | --- | --- | --- | --- |
| SUB2594650 | SAMN06771400 | NEHD00000000 | Pseudomonas sp. R9(2017) | 45 | 5.99 |
| SUB2594650 | SAMN06771381 | NEHW00000000 | Pseudomonas sp. R28(2017) | 44 | 5.99 |
| SUB2594650 | SAMN06771371 | NEIG00000000 | Pseudomonas sp. R17(2017) | 47 | 5.99 |
| SUB2594650 | SAMN06771368 | NEIJ00000000 | Pseudomonas sp. R14(2017) | 51 | 5.99 |
| SUB2594650 | SAMN06771366 | NEIL00000000 | Pseudomonas sp. R11(2017) | 47 | 5.99 |
| SUB2594650 | SAMN06771365 | NEIM00000000 | Pseudomonas sp. R10(2017) | 67 | 5.98 |
| SUB2594650 | SAMN06771364 | NEIN00000000 | Pseudomonas sp. B9(2017) | 62 | 5.56 |
| SUB2594650 | SAMN06771363 | NEIO00000000 | Pseudomonas sp. B8(2017) | 56 | 5.89 |
| SUB2594650 | SAMN06771360 | NEIR00000000 | Pseudomonas sp. B5(2017) | 50 | 5.88 |
| SUB2594650 | SAMN06771359 | NEIS00000000 | Pseudomonas sp. B4(2017) | 64 | 5.92 |
| SUB2594650 | SAMN06771340 | NEJL00000000 | Pseudomonas sp. B24(2017) | 64 | 5.93 |
| SUB2594650 | SAMN06771339 | NEJM00000000 | Pseudomonas sp. B23(2017) | 52 | 5.93 |
| SUB2594650 | SAMN06771338 | NEJN00000000 | Pseudomonas sp. B22(2017) | 43 | 5.61 |
| SUB2594650 | SAMN06771334 | NEJR00000000 | Pseudomonas sp. B19(2017) | 57 | 5.56 |
| SUB2594650 | SAMN06771333 | NEJS00000000 | Pseudomonas sp. B18(2017) | 58 | 5.56 |
| SUB2594650 | SAMN06771325 | NEJU00000000 | Pseudomonas sp. B10(2017) | 51 | 5.65 |
| SUB2594650 | SAMN06771327 | NEJW00000000 | Pseudomonas sp. B12(2017) | 66 | 5.95 |
| SUB2594650 | SAMN06771328 | NEJX00000000 | Pseudomonas sp. B13(2017) | 61 | 5.95 |
| SUB2594650 | SAMN06771329 | NEJY00000000 | Pseudomonas sp. B14(2017) | 64 | 5.93 |
