# Supplementary material for: Genomic and metabolic differences between Pseudomonas putida populations inhabiting sugarcane rhizosphere or bulk soil
Source: PLoS One. 2019 Oct 3;14(10):e0223269. doi: 10.1371/journal.pone.0223269 (PMC6776310; doi:10.1371/journal.pone.0223269)
Supplement: S2 Table — (DOCX) [file pone.0223269.s003.docx]

| Assembly Accession | Type Strain | Genome Size (Mbp) |
| --- | --- | --- |
| [GCA_000621265.1](https://www.ncbi.nlm.nih.gov/assembly/GCA_000621265.1) | *P. fulva* DSM 17717 | 4.77 |
| \|  \| [GCA_000425745.1](https://www.ncbi.nlm.nih.gov/assembly/GCA_000425745.1) \| \| --- \| --- \| | *P. cremoricolorata* DSM 17059 | 4.66 |
| [GCA_000425805.1](https://www.ncbi.nlm.nih.gov/assembly/GCA_000425805.1) | *P. vranovensis* DSM 16006 | 5.70 |
| [GCA_000730585.1](https://www.ncbi.nlm.nih.gov/assembly/GCA_000730585.1) | *P. japonica* NBRC 103040 | 6.66 |
| [GCA_000761155.1](https://www.ncbi.nlm.nih.gov/assembly/214341) | *P. rhizosphaerae* DSM 16299 | 4.69 |
| [GCA_000621225.1](https://www.ncbi.nlm.nih.gov/assembly/GCA_000621225.1) | *P. mosselii* DSM 17497 | 6.26 |
| [GCA_900110655.1](https://www.ncbi.nlm.nih.gov/assembly/GCA_900110655.1) | *P. soli* LMG 27941 | 5.64 |
| [GCA_000026105.1](https://www.ncbi.nlm.nih.gov/assembly/GCA_000026105.1) | *P. entomophila* L48 | 5.89 |
| [GCA_000425785.1](https://www.ncbi.nlm.nih.gov/assembly/GCA_000425785.1) | *P. taiwanensis* DSM 21245 | 5.41 |
| \| [GCA_900102675.1](https://www.ncbi.nlm.nih.gov/assembly/GCA_900102675.1) \|  \| \| --- \| --- \| | *P. guariconensis* LMG 27394 | 5.08 |
| [GCA_000425765.1](https://www.ncbi.nlm.nih.gov/assembly/GCA_000425765.1) | *P. parafulva* DSM 17004 | 4.96 |
| [GCA_000731675.1](https://www.ncbi.nlm.nih.gov/assembly/199151) | *P. capeferrum* WCS358 | 5.94 |
| [GCA_000688275.1](https://www.ncbi.nlm.nih.gov/assembly/GCA_000688275.1) | *P. plecoglossicida* DSM 15088 | 5.35 |
| [GCA_000412675.1](https://www.ncbi.nlm.nih.gov/assembly/GCA_000412675.1) | *P. putida* NBRC 14164 | 6.16 |
| [GCA_000621245.1](https://www.ncbi.nlm.nih.gov/assembly/GCA_000621245.1) | *P. monteilii* DSM 14164 | 6.31 |
